# Viral Load, Clinical Disease Severity and Cellular Immune Responses in Primary Varicella Zoster Virus Infection in Sri Lanka

**DOI:** 10.1371/journal.pone.0003789

**Published:** 2008-11-21

**Authors:** Gathsaurie Neelika Malavige, Louise Jones, S. D. Kamaladasa, A. Wijewickrama, S. L. Seneviratne, Antony P. Black, Graham S. Ogg

**Affiliations:** 1 MRC Human Immunology Unit, Oxford Biomedical Research Centre, Weatherall Institute of Molecular Medicine, John Radcliffe Hospital, Oxford, United Kingdom; 2 Department of Medicine, Faculty of Medical Sciences, University of Sri Jayawardanapura, Gangodawila, Nugegoda, Sri Lanka; 3 Infectious Diseases Hospital, Colombo, Sri Lanka; 4 Department of Clinical Immunology, Imperial College AHSC (St Mary'sHospital), London, United Kingdom; 5 Department of Microbiology, Faculty of Medical Sciences, University of Sri Jayawardanapura, Gangodawila, Nugegoda, Sri Lanka; 6 Department of Dermatology, Churchill Hospital, Oxford, United Kingdom; Centre de Recherche Public-Santé, Luxembourg

## Abstract

**Background:**

In Sri Lanka, varicella zoster virus (VZV) is typically acquired during adulthood with significant associated disease morbidity and mortality. T cells are believed to be important in the control of VZV replication and in the prevention of reactivation. The relationship between viral load, disease severity and cellular immune responses in primary VZV infection has not been well studied.

**Methodology:**

We used IFNγ ELISpot assays and MHC class II tetramers based on VZV gE and IE63 epitopes, together with quantitative real time PCR assays to compare the frequency and phenotype of specific T cells with virological and clinical outcomes in 34 adult Sri Lankan individuals with primary VZV infection.

**Principal Findings:**

Viral loads were found to be significantly higher in patients with moderate to severe infection compared to those with mild infection (p<0.001) and were significantly higher in those over 25 years of age (P<0.01). A significant inverse correlation was seen between the viral loads and the *ex vivo* IFNγ ELISpot responses of patients (P<0.001, r = −0.85). VZV-specific CD4+ T cells expressed markers of intermediate differentiation and activation.

**Conclusions:**

Overall, these data show that increased clinical severity in Sri Lankan adults with primary VZV infection associates with higher viral load and reduced viral specific T cell responses.

## Introduction

Primary infection with varicella zoster virus (VZV) results in chickenpox, which is usually a benign self-limiting illness, characterized by fever and a generalized pruritic vesicular rash. However, in certain groups of individuals such as neonates, adults, pregnant women and immunosuppressed individuals, it may cause severe disease and can sometimes be fatal. Adults are 9 to 15 times more likely to be hospitalized [1] and 25 times more likely than children to die from varicella [2]. Varicella associated complications such as pneumonia are more common among adults than children [3]. Primary varicella in immunosuppressed individuals may result in visceral dissemination, multi organ failure and death [4]–[6].

Although varicella infections occur worldwide, there are marked differences in its epidemiology in tropical and temperate climates. In temperate climates, chickenpox is a common childhood illness and seropositivity rates range from 53% to 100% in 5 year olds, and in 20–30 years olds it is typically greater than 80% [7]–[9]. In contrast, in the tropics, due to the lower incidence of VZV infection among children, it more commonly affects adults [10]–[12], thus resulting in significant morbidity and mortality. In Sri Lanka 56.2% of females of child bearing age were antibody negative for VZV [13]. Approximately 1000 patients with VZV infections are admitted to just one infectious diseases hospital in Colombo in Sri Lanka each year [14] and many patients develop complications with an overall mortality rate of 4.2% [14]. VZV is therefore a significant health problem in Sri Lanka and understanding of the immunological correlates of disease will be important for new vaccine and treatment developments.

VZV infects many cell types in the host during acute infection, including T cells, B cells, monocytes and dendritic cells [15]–[17]. Infection of T cells by the virus is thought to be one of the main mechanisms by which the virus disseminates, subsequently infecting keratinocytes and other cells [17], [18]. During the viraemic phase, which is thought to be highly cell associated [19], VZV is believed to predominantly infect T cells [20], [21]. In acute primary VZV infection, viral loads in children have been reported in the range of 1 to 5000 viral copies per 10^5^ PBMCs [22], [23] and 100 to 10000 per ml of blood [24]. In many infectious diseases, the degree of viraemia is thought to be associated with severity of clinical disease [25]–[27], however, the correlation of the degree of VZV viraemia with clinical disease severity has not been investigated in patients with acute primary VZV infection.

T cell responses are believed to be important in controlling the virus and preventing viral reactivation. Virus specific proliferative T cell responses were found to be impaired in immunosuppressed individuals with severe disease [28], [29] and proliferative T cell responses in the first 72 hours since the onset of symptoms were shown to be associated with milder disease [30]. Interestingly, VZV specific antibody titres did not seem to correlate with clinical disease severity [30], [31]. These studies suggest that a strong VZV-specific T cell response early in infection may protect individuals from severe disease. However, this possibility and the associations between the frequency and functional T cell responses with viral load in acute primary VZV infection have not been investigated in detail.

In order to further understand the host pathogen interactions during primary VZV infection, we set out to investigate the degree of viral load, and phenotype and functionality of T cell responses in relation to clinical disease severity in a cohort of adult patients with acute primary VZV infection.

## Materials and Methods

### Subjects

Fresh heparinized venous blood samples were obtained from 34 adult individuals with acute primary varicella infection who were admitted sequentially to the Infectious Diseases Hospital in Sri Lanka. Ethics was obtained from the Ethics Committee of University of Sri Jayawardanapura, Sri Lanka and the Oxfordshire Research Ethics Committee, UK. It is common practice in Sri Lanka that even individuals with mild disease will attend hospital and will frequently be hospitalised. Written consent was gained from all donors. Mean age of the donors was 32.8 (SD±1.7) years and the mean duration of symptoms since the onset of rash at the time of taking the first blood sample 4.5 days (SD±1.7, median 4.5, range 3 to 7 days).

The clinical disease severity was assessed by using the severity scale defined by Vazquez M *et al.* which classifies severity of infection based on the number and character of the lesions, presence or absence of fever, systemic signs and also the subjective assessment of the patient [32]. Accordingly, 12 had mild infection, and 22 had moderate to severe infection. 13 patients had more >500 skin lesions and 7 had <50 skin lesions. The remaining 14 patients had skin lesions in the range of 50 to 500.

### Ex vivo ELISpot assays and intracellular cytokine assays

Peripheral blood mononuclear cells (PBMC) were obtained from fresh heparinized blood by Ficoll-Hypaque density gradient centrifugation. They were then resuspended in RPMI 1640 plus 10% fetal calf serum (FCS) for *ex vivo* ELISpot assays and *ex vivo* ICS assays and in RPMI 1640 plus 10% human serum for cell cultures.

*Ex vivo* Elispot assays were performed as previously described [33]. Briefly, ELISpot plates (Millipore Corp., Bedford, Massachusetts, USA) were coated with anti-human IFNγ antibody overnight (Mabtech AB, Nacka, Sweden). The plates were washed six times with RPMI 1640 and incubated for 1 hour with RPMI-1640 and 10% FCS. 0.1×10^6^ PBMC were added to a final volume of 200 µl. The live attenuated varicella zoster vaccine (Varilrix: GlaxoSmithKline) was added to a final concentration of 10*4 pfu/ml. All peptides were tested in duplicate. PHA was included as a positive control and an irrelevant peptide was included as a negative control. Positive responses were defined as mean plus 3 standard deviations of the irrelevant peptide responses. The plates were incubated overnight at 37°C and 5% CO_2_. The cells were removed and the plates developed with a second biotinylated Ab to human IFNγ and washed a further six times. The plates were developed with streptavidin-alkaline phosphatase (Mabtech AB) and colorimetric substrate, and the spots enumerated using an automated ELISpot reader. Background (cells plus media) was subtracted and data expressed as number of spot-forming units (SFU) per 10^6^ PBMC.

To determine IFNγ production, *ex vivo*- PBMC or T-cell lines were stimulated at 1×10^6^ to 2×10^6^/ml in RPMI 1640 plus 10% FCS with the VZV live attenuated vaccine for 16 hours according to manufacturers instructions in the presence of Brefeldin A (BD GolgiStop™). Cells were washed and stained with anti CD3 (FITC), anti CD4 (PerCP) (BD Biosciences) and anti CD8 (PE). Cells were then permeabilized and fixed with Cytofix/Cytoperm (BD Biosciences) and then stained for intracellular IFNγ (APC). Cells were acquired on a CyAn™ (DakoCytomation) and analysed using FlowJo software.

### Tetramer and phenotypic staining and flow cytometry

DRB1*1501 iTAg MHCII tetramers were purchased from Beckman Coulter. DRB1*1501 tetramer was complexed to VZV gE peptide 54 (aa531–545; TSPLLRYAAWTGGLA) [42] or VZV IE63 peptide 24 (aa229–243; QRAIERYAGAETAEY) [38]. These peptides were chosen on the basis that they were commonly recognised in previous studies [38], [42]. Unless stated otherwise, cell lines and PBMC were incubated with 2 µg/ml HLA class II tetramer for 60 min at 37°C in RPMI-1640 and 10% human serum. We analyzed the tetramer expression within the CD4^+^T cell subset by gating on the lymphocytes and excluding B cells, monocytes and dead cells (via probe positive population).

The cell surface marker Abs CD4-pacific blue (Biolegend), CD14-PerCP, C19-PerCP and 7-aminoactinomycin D (7-AAD) (all BD Pharmingen) were added for 20 minutes at room temperature. For phenotypic analysis of tetramer-positive CD4^+^ T cells, antibodies to CD38 (APC); CD62L (APC); CCR7 (Pe-Cy7) and CLA (FITC) were added with the other surface antibodies. Stained cells were washed with PBS, and fixed in 0.5% PBS/formaldehyde. Cells were acquired on a CyAn™ (DakoCytomation) and analysed using FlowJo software.

### Quantitative real time PCR

Quantitative real time PCR was performed as previously described using the ABI Prism 7700 sequence detector system [34], [35]. Forward primers (5′-CGTACACGTATTTTCAGTCCTCTTC-3′) and reverse primers (5′-GGCTTAGACGTGGAGTTGACA-3′) for VZV ORF29 and a probe (5′-(FAM)CCCGTGGAGCGCGTCGAAA(TAMRA)-3′) were used [35].

Standard curves for the viral gene were generated by using a plasmid with serial 10 fold dilutions. The plasmid was generated in-house by inserting viral DNA extracted from the VZV live attenuated vaccine in to a pCR 2.1-TOPO® vector. Briefly, the PCR fragment amplified from the ORF29 was cloned in to the vector using one Shot DH5α™-T1 chemically competent *E.coli* according to the manufacturers instructions (Invitrogen, United Kingdom). Clones containing the vector and the insert were isolated and plasmid DNA was extracted from the competent E. coli using the QIAprep Spin Miniprep Kit according to the manufacturer's instructions. Digestion of plasmid DNA was performed to determine the molecular weight of the DNA fragment which was inserted into the vector and subsequently sequenced to ensure that the insert was of the correct sequence.

### Detection of viral loads in patients with primary VZV infection

Briefly, viral DNA was extracted from whole EDTA blood samples from the patients using Gentra PureGene Kit (D5000). All PCRs were performed in triplicate. The standard curves used for data analysis all had a correlation coefficient exceeding 0.985. PCR mixtures were amplified for 2 min at 50°C, 10 min at 95°, 60 cycles of 15 s at 95°C and 1 min at 60°C. Data was analyzed using the PE Applied Biosystems- sequence detection systems 1.5. The real-time fluorescence values were measured by the quantity of a reporter dye FAM released during amplification. The threshold cycle value (Ct) for each reaction reflects the cycle number in which the fluorescence exceeds the threshold. The threshold limit was set in the linear phase of exponential amplification after viewing the log linear view of the amplification plot. All samples were done in triplicate and expressed as median.

## Results

### Viral load and clinical disease severity in adult patients with primary VZV infection

**Figure 1:**
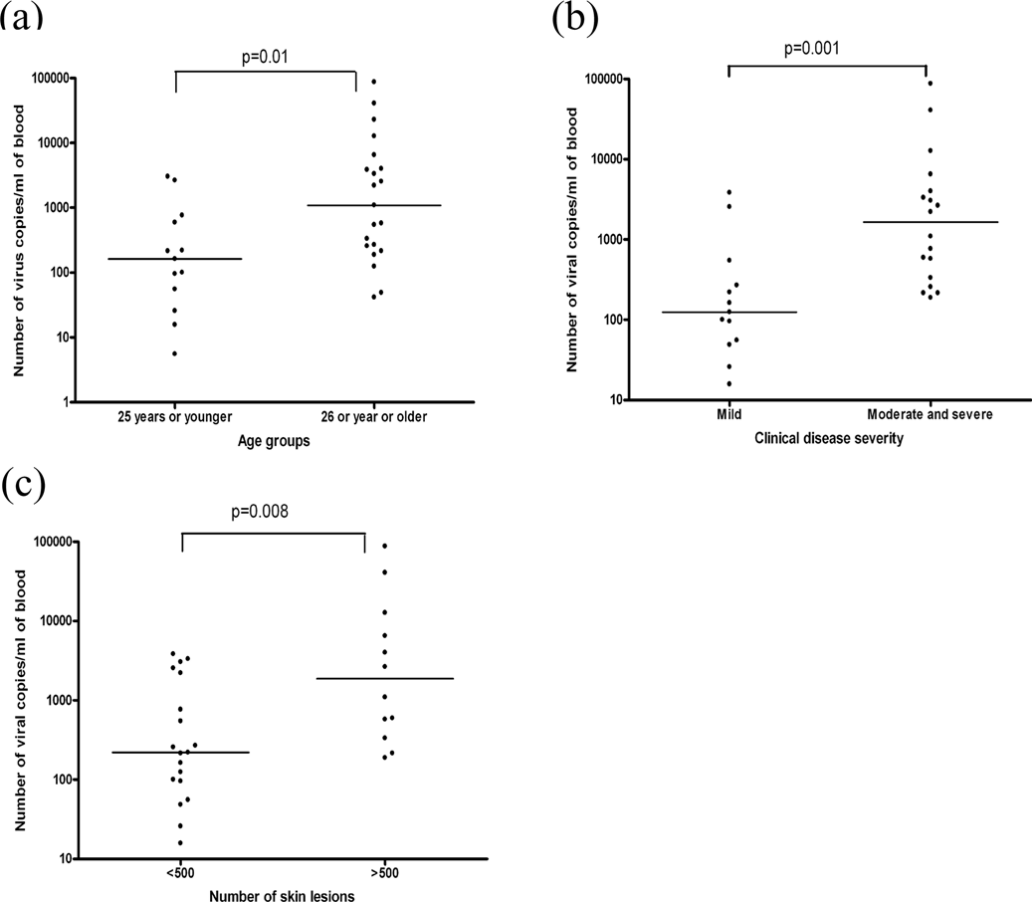
Relation of clinical characteristics to viral load. (A) Viral loads in patients with primary VZV who were 25 years or younger compared to older individuals. (B) Quantity of viral copies in the peripheral blood of patients with varying severity of primary VZV infection. (C) Quantity of viral copies in patients with primary VZV infection with different numbers of skin lesions. Line shows median.

Tables 1 and 2 document the clinical characteristics of the cohort. We confirmed a significant positive correlation with clinical disease severity and the age of the patients in our cohort (Spearman's r = 0.47, P = 0.0067). However, we showed that peripheral blood viral loads were significantly higher in those aged over 25 years (P = 0.01) when compared to those between 18 to 25 years (Fig 1A). In addition, a significant correlation was observed between the age of the patients and the viral loads (P = 0.02, Spearman's r = 0.39).

**Table 1:** Clinical characteristics of cohort.

Donor	Age	Duration of symptoms prior to blood sample	Total severity score	Complications
SLCP01	42	3	15	Bacterial meningitis
SLCP02	31	5	11	
SLCP03	19	2	7	
SLCP04	79	3	7	
SLCP05	31	5	15	
SLCP06	20	9	6	AML
SLCP07	19	4	6	
SLCP08	40	5	11	
SLCP09	43	2	10	
SLCP10	13	4	7	Infected lesions
SLCP11	37	3	20	Nephrotic syn. (steroids)
SLCP12	21	2	10	
SLCP14	31	5	11	
SLCP14	54	7	11	
SLCP15	58	5	6	
SLCP16	31	6	9	
SLCP17	50	14	9	VZV Cerebellitis
SLCO18	29	5	6	
SLCP19	46	6	12	
SLCP20	25	5	11	
SLCP21	32	3	7	
SLCP22	46	5	25	VZV associated Severe pneumonia
SLCP23	58	7	13	
SLCP24	25	6	15	SLE (steroids)
SLCP25	19	4	7	
SLCP26	27	3	6	
SLCP27	29	3	12	
SLCP28	17	5	9	
SLCP29	28	4	6	
SLCP30	19	4	10	
SLCP31	24	8	12	
SLCP32	24	3	8	
SLCP33	17	3	3	
SLCP34	32	4	9	Melanoma

**Table 2:** Relation of age to number of lesions.

Age	<50	51–100	101–500	>500
16–25	4	4	2	1
26–40	1	3	2	7
>41	2	2	1	5

We next went on to determine the viral loads in relation to varying clinical disease severity. Indeed the viral loads in patients with moderate and severe disease (median 1,652 viral copies/ml blood) were significantly higher (P<0.001) than in those with mild disease (median 124 viral copies/ml blood) (Fig 1B). A significant correlation (P = 0.0005, Spearmans r = 0.56) was also observed between the clinical disease severity and the number of viral copies in these patients.

Within the disease severity scale, a significant emphasis is given to the number of skin lesions. We compared the viral loads in patients with different numbers of skin lesions. The number of viral copies was significantly higher in patients with >500 skin lesions, when compared to those with <500 skin lesions (P = 0.008) (Fig 1C). Thus, the presence of >500 skin lesions was an indicator of the presence of a higher viral load in patients with acute infection.

Having established that clinical disease severity and VZV viral load were associated with an increase in age and also that a higher viral load was associated with more severe clinical disease severity and the presence of >500 skin lesions, we went on to determine whether differences in T cell responses might associate with viral load and disease severity.

### T cell responses in patients with primary VZV infection

We initially proceeded to investigate the functional T cell responses by using *ex vivo* IFNγ ELISpot assays with peripheral blood mononuclear cells (PBMC) derived from twelve individuals. Although varied a frequency of *ex vivo* IFNγ responses to the VZV live vaccine were seen in these patients, 4 patients had no detectable VZV-specific IFNγ production despite the presence of symptoms for 3 to 5 days. VZV-specific IFNγ responses were higher in patients with mild infection (median 622 spot forming units/million cells) than in patients with moderate/severe disease (median 40 spot forming units/million cells). Patients with <500 skin lesions had significantly higher (median 615 spot forming units/million cells) VZV-specific IFNγ responses (P = 0.02) than patients with >500 skin lesions (median 30 spot forming units/million cells).

**Figure 2:**
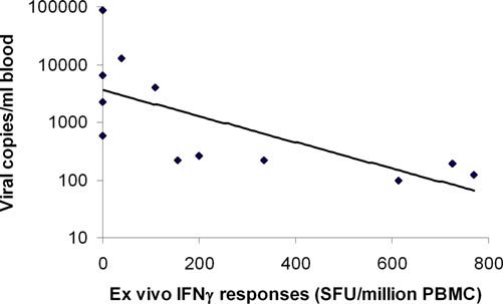
Inverse correlation of viral loads with *ex vivo* IFNγ ELISpot responses of patients with acute varicella infection. Spearman's r = −0.85, p<0.001.

As patients with more severe disease appeared to have lower VZV-specific T cell responses, we then went on to determine if there was any association between the viral loads in these patients and VZV-specific IFNγ responses. A significant negative correlation was observed between the viral loads and VZV-specific *ex vivo* IFNγ responses in such individuals (Spearman's r = −0.85, P<0.001) (Fig. 2).

**Figure 3:**
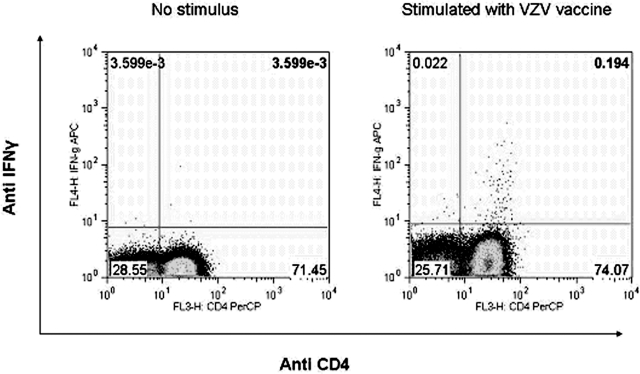
CD4+ T cells represent the main source of VZV-specific IFNγ production during acute infection. Example of *ex vivo* intracellular cytokine analysis of PBMC of a patient with acute varicella infection. The VZV vaccine was used as the antigenic stimulus. Cells are gated on CD3+.

We have previously mapped several VZV CD4+ T cell epitopes in VZV glycoproteins gI and gE and immediate early proteins IE4 and IE63 [36]–[38]. We and others have found that in healthy VZV immune donors the majority of the VZV-specific T cell responses were from the CD4+ subset of T cells [36], [37], [39]. Using *ex vivo* intra cellular cytokine assays we observed that even in acute primary varicella infection, IFNγ production was predominantly from the CD4+ subset of T cells within the peripheral blood (Fig 3). However, clearly it will be important to investigate other sites such as the skin, to determine whether VZV-specific CD8+ T cells are preferentially sequestered at particular sites.

### Analysis of the frequency and phenotype of gE and IE63 DRB1*1501 tetramer specific responses in patients with acute VZV infection

**Figure 4:**
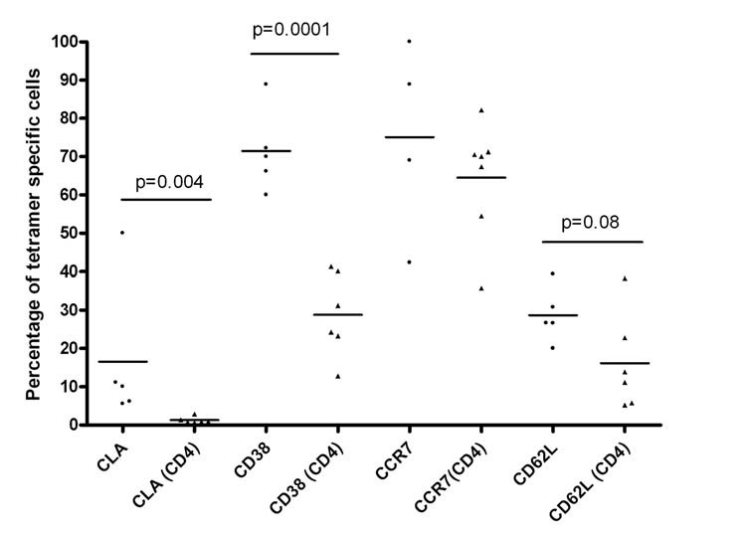
VZV-specific CD4+ T cells phenotypic analysis. Comparison of the expression of CLA (cutaneous lymphocyte associated antigen-1), lymph node homing markers and activation markers on the tetramer-binding cells (circles) and total CD4+ population (triangles). Cells are gated on CD4+ Via probe^−^CD14^−^CD19^−^ cells.

Following analysis of the functional responses of VZV-specific T cells in patients with acute VZV infection using whole viral vaccine as immunogen, we then proceeded to investigate the frequency and phenotype of common gE and IE63 DRB1*1501 epitope specific CD4+ T cell responses in these patients. This approach allows the detection of antigen specific T cells without the requirement of a functional outcome. In our cohort, 8 patients were of the appropriate DRB1*1501 HLA genotype. Frequencies of gE DRB1*1501 tetramer specific T cells ranged from 0 to 0.097% (median 0.0157% of CD4+ T cells) while the frequencies of IE63 DRB1*1501 tetramer specific T cells ranged from 0 to 0.092% (median 0.007% of CD4+ T cells). During acute infection VZV-tetramer specific T cells and showed an activated phenotype with preferential expression of skin homing receptors compared to the total CD4+ population (Fig 4). Cutaneous lymphocyte associated antigen (CLA) was expressed by significantly more of the tetramer-positive cells compared to the total CD4+ population (median 10% and 0.9% respectively; P<0.01). Furthermore CD38 was also expressed by significantly more of the tetramer-positive cells than the total CD4+ population (70% and 28% respectively; P<0.001). There was no significant difference in CCR7 expression, but the expression of CD62L was markedly higher in the tetramer-positive population than in the total CD4+ population (26.7% and 12.4% respectively) but this did not reach statistical significance. Clearly the total CD4+ T cell population will contain T cells specific for other VZV epitopes and therefore phenotypic comparisons should be interpreted with caution. Nevertheless, the functional T cell responses elicited are not high (maximum 770 spot forming units per million PBMC) and it is therefore likely that the vast majority of the peripheral blood CD4+ T cells are not specific for VZV.

## Discussion

It is well established that adults are more likely to develop severe disease and complications during primary VZV infection [1], [3] and our results confirm that the clinical disease severity score increases significantly with advancing age. In this study, we have extended these findings to show that there is a positive correlation between age and peripheral blood viral load during disease.

Patients with >500 skin lesions had significantly higher viral loads than patients with fewer lesions. Therefore, the presence of >500 skin lesions appears to be a good clinical indicator of high viral loads in peripheral blood. This was in contrast to observations in herpes zoster (HZ), where rash severity does not correlate with the extent of viraemia in acute HZ. Rather the viral load in HZ appeared to be associated with age and immune status of the individual [34].

A large variation was observed in VZV-specific IFNγ responses in patients with acute infection. Patients with milder disease had higher VZV-specific IFNγ responses than those with more severe disease (p = 0.05). Moreover, patients with a >500 skin lesions had lower IFNγ producing VZV-specific T cells than those with fewer lesions. However, the virus specific IFNγ responses were significantly lower than those observed in many other acute viral infections [40], [41] despite evidence of detectable virus in the blood.

We observed a significant inverse correlation between rapid IFNγ production by VZV-specific T cells and viral load. Such an inverse correlation suggests that IFNγ-producing T cells may be important for early control of viral replication. In order to examine responses at the epitope-specific level, we also used HLA-peptide tetrameric complexes to identify the presence of specific T cells. The tetramer-positive T cell phenotype was that of intermediate differentiation with evidence of recent activation. Furthermore 10% of the cells expressed CLA and thus would be likely to have skin homing capacity. It will clearly be important to examine other markers putatively associated with skin homing such as CCR4 and CCR10. CCR7 expression was maintained in the majority of cells suggesting that these cells retained the capacity for ongoing lymph node migration, likely to be important for control of a virus associated with viraemia. The frequencies of gE and IE63 epitope-specific T cells were higher in patients with acute infection compared to previous studies in healthy immune donors where the median levels were 0.003% and 0.006% respectively [38], [42]. Frequencies of tetramer-binding CD4+ T cells were however in a similar range to those observed during acute parvovirus B19 and hepatitis C infections [43]–[45]. Nevertheless the inverse association between functional VZV-specific T cell responses and both viral load and disease severity argues in support of a potential role of such levels of T cells in the control of viral replication. In addition, although CD8+ T cells are thought to play a major role in controlling acute herpes virus infection, virus specific CD4+ T cells have also been shown to possibly play an important role. For instance, it was observed that IFNγ secreting CD4+ T cell responses developed earlier in patients with asymptomatic CMV infection than in patients with symptomatic infection and were associated with clearance of the virus. Moreover, despite the presence of specific antibody and CMV-specific CD8+ T cells, clearance of virus in symptomatic patients only occurred after emergence of IFNγ secreting CD4+ T cells [46], [47]. However, it is also possible that as patients with more severe clinical disease had a higher number of skin lesions, virus specific T cells could be localized in the skin thereby reflecting a lower number of specific T cells in the peripheral blood. We have observed that the peripheral blood response to VZV is dominated by CD4+ T cells during acute infection, which is analogous to observations in healthy immune donors [36]–[39]. It is not clear whether such relative paucity of viral-specific CD8+ T cells reflects impaired class I antigen presentation or selective targeting of VZV-specific CD8+ T cells by the virus. VZV inhibits the IFNγ mediated induction of class I [17], [48], but many other viruses employ such an immune evasion strategy (eg CMV, HIV) and yet CD8+ T cell responses are readily detected to these viruses. Understanding such mechanisms will clearly be important for identification of novel therapeutic options relevant to VZV and other herpes viruses.

It will clearly be important to undertake similar analyses in other populations in order to establish whether the findings are consistent between, for example, tropical and temperate climates. Whilst possible explanations for differences in epidemiology are debated and may include viral, climate and host factors, acquisition of primary disease during adulthood consistently associates with more severe disease in diverse populations. It will also be important to undertake longitudinal analyses, both in the presence and absence of anti-viral therapy in order to characterise the T cell correlates of disease and viral load over time.

In summary, these data provide a mechanistic link between clinical disease severity and T cell responses during primary infection with VZV. The presence of >500 skin lesions was a useful and simple clinical indication of high viral loads and impaired VZV-specific functional T cell responses. Individuals with maintained IFNγ effector function of VZV-specific T cells had lower viral loads and lower disease severity scores.
